# Prediction of pre-eclampsia at St. Mary's hospital lacor, a low-resource setting in northern Uganda, a prospective cohort study

**DOI:** 10.1186/s12884-023-05420-z

**Published:** 2023-02-08

**Authors:** Silvia Awor, Benard Abola, Rosemary Byanyima, Christopher Garimoi Orach, Paul Kiondo, Dan Kabonge Kaye, Jasper Ogwal-Okeng, Annettee Nakimuli

**Affiliations:** 1 Department of Obstetrics and Gynaecology, Faculty of Medicine, Gulu University, P.O.Box 166, Gulu, Uganda; 2 Department of Mathematics, Faculty of Science, Gulu University, P.O.Box 166, Gulu, Uganda; 3 Department of Radiology, Mulago National Referral Hospital, PO Box 7051, Kampala, Uganda; 4 Department of Community Health, School of Public Health, College of Health Sciences, Makerere University, Kampala City, Uganda; 5 Department of Obstetrics and Gynaecology, School of Medicine, College of Health Sciences, Makerere University, P.O.Box 7062, Kampala, Uganda; 6 Department of Pharmacology, Lira University, Lira, Uganda

**Keywords:** Risk prediction, Uterine artery Doppler indices, Maternal history, Blood tests, Pre-eclampsia, Uganda, Africa

## Abstract

**Background:**

Pre-eclampsia is the second leading cause of maternal death in Uganda. However, mothers report to the hospitals late due to health care challenges. Therefore, we developed and validated the prediction models for prenatal screening for pre-eclampsia.

**Methods:**

This was a prospective cohort study at St. Mary's hospital lacor in Gulu city. We included 1,004 pregnant mothers screened at 16–24 weeks (using maternal history, physical examination, uterine artery Doppler indices, and blood tests), followed up, and delivered. We built models in RStudio. Because the incidence of pre-eclampsia was low (4.3%), we generated synthetic balanced data using the ROSE (Random Over and under Sampling Examples) package in RStudio by over-sampling pre-eclampsia and under-sampling non-preeclampsia. As a result, we got 383 (48.8%) and 399 (51.2%) for pre-eclampsia and non-preeclampsia, respectively. Finally, we evaluated the actual model performance against the ROSE-derived synthetic dataset using K-fold cross-validation in RStudio.

**Results:**

Maternal history of pre-eclampsia (adjusted odds ratio (aOR) = 32.75, 95% confidence intervals (CI) 6.59—182.05, *p* = 0.000), serum alkaline phosphatase(ALP) < 98 IU/L (aOR = 7.14, 95% CI 1.76—24.45, *p* = 0.003), diastolic hypertension ≥ 90 mmHg (aOR = 4.90, 95% CI 1.15—18.01, *p* = 0.022), bilateral end diastolic notch (aOR = 4.54, 95% CI 1.65—12.20, *p* = 0.003) and body mass index of ≥ 26.56 kg/m^2^ (aOR = 3.86, 95% CI 1.25—14.15, *p* = 0.027) were independent risk factors for pre-eclampsia. Maternal age ≥ 35 years (aOR = 3.88, 95% CI 0.94—15.44, *p* = 0.056), nulliparity (aOR = 4.25, 95% CI 1.08—20.18, *p* = 0.051) and white blood cell count ≥ 11,000 (aOR = 8.43, 95% CI 0.92—70.62, *p* = 0.050) may be risk factors for pre-eclampsia, and lymphocyte count of 800 – 4000 cells/microliter (aOR = 0.29, 95% CI 0.08—1.22, *p* = 0.074) may be protective against pre-eclampsia. A combination of all the above variables predicted pre-eclampsia with 77.0% accuracy, 80.4% sensitivity, 73.6% specificity, and 84.9% area under the curve (AUC).

**Conclusion:**

The predictors of pre-eclampsia were maternal age ≥ 35 years, nulliparity, maternal history of pre-eclampsia, body mass index, diastolic pressure, white blood cell count, lymphocyte count, serum ALP and end-diastolic notch of the uterine arteries. This prediction model can predict pre-eclampsia in prenatal clinics with 77% accuracy.

## Introduction

Pre-eclampsia (PE), a pregnancy syndrome, is characterised by hypertension and proteinuria [1, 2]. Approximately 90 per cent of cases present in the late preterm (≥ 34 weeks) period and have good maternal and fetal outcomes [2, 3]. However, 10 per cent of cases who have an early presentation (< 34 weeks) have more severe disease and carry the additional high risks associated with preterm birth [3, 4]. In addition, mothers with a history of pre-eclampsia are at increased risk for developing the cardiovascular and renal disease [2, 3].

Risk factors of PE include low socio-economic status, nulliparity, multiple pregnancies, obesity, chronic hypertension, being a woman of African descent, previous maternal or family history of pre-eclampsia, and maternal age ≥ 35 years [5–7]. In addition, high second-trimester artery Doppler resistive index, pulsatility index, and end-diastolic notch are known risk factors for pre-eclampsia [8, 9]. Early diagnosis and delivery of the fetus is the only known treatment, thus necessitating the need for prediction models of this disorder.

De Kat et al. [10] summarised risk factors and models for predicting pre-eclampsia. Black race stood out as a significant risk factor in all the studies where the communities had a mixed race, insinuating that the predominantly black Ugandan communities are at high risk for pre-eclampsia. Studies by Al-Rubaie et al. [11] achieved the highest area under the curve (AUC) for predicting pre-eclampsia at 76% using maternal history. Gallo et al. [12] screened for PE using maternal history and mean arterial pressure (MAP) at a false-positive rate of 10%; their detection rate of total pre-eclampsia was 49.3%. Jhee et al. [13] used laboratory tests (serum urea, aspartate aminotransferase (AST), alanine transaminase (ALT), creatinine, and haemoglobin levels) to predict pre-eclampsia. They got an area under the curve (AUC) above 57%. Delic et al. added uric acid, urea thrombocytes, hematocrit, AST, and leukocytes to the regression model and correctly classified 83.8% of patients with pre-eclampsia [14]. Yucel et al. [15] predicted pre-eclampsia using mean platelet volume (MPV) with an AUC of 64.1% and plateletcrit (PCT) with an AUC of 71.2%.

Antwi et al. [16] reviewed prediction models for pre-eclampsia between 2000 and 2019 and found diverse prediction accuracy ranging from 45 – 95% in the different regions of the world. After observing the wide variation in prediction rates of pre-eclampsia and consistently having the black race as a risk factor for pre-eclampsia, we developed and validated risk prediction models based on maternal characteristics from northern Uganda.

## Methods

The research was a prospective cohort study at St. Mary's Hospital Lacor. This hospital is a private, not-for-profit hospital founded by the Catholic Church. It is located six kilometres west of Gulu city along Juba Road in Gulu district (Longitude 30 – 32 degrees East and Latitude 02 – 04 degrees North). St. Mary's Hospital Lacor is one of the teaching hospitals of Gulu University with a bed capacity of 482. It is staffed by specialists, medical officers, midwives, nurses, laboratory and radiology staff, and support and administrative staff. The hospital receives about three thousand six hundred antenatal mothers and conducts about six thousand deliveries annually [17]. Some mothers go to the hospital for delivery without prenatal care; others are referred from smaller health units. The mothers pay five thousand Uganda shillings (Ugx 5,000/ =) ($1.5) as the cost per visit. This cost is often waived for most mothers who cannot afford it. Normal labour and delivery cost fifteen thousand (Ugx 15,000/ =) (about $4.50), and Caesarean section costs twenty-five thousand (Ugx 25,000/ =) (about $7.5) Uganda shillings.

Using Yamane's 1967 formula [18] for calculating sample size for cohort studies using finite population size: St. Mary's hospital Lacor receives approximately three thousand six hundred antenatal mothers annually. Since my study duration was 24 months, the limited population we could access was about 7,200 mothers.

**Table:** 

Yamane 1967 formula: Sample size	*n* = N / 1 + Ne^2^
Where N is the finite population size	of 7,200 mothers
The margin of error (e)	05%
Therefore	*n* = 7,200 / 1 + 7,200(0.05)^2^
	*n* = 379

We doubled the sample size to take care of loss to follow-up. We targeted all pregnant women attending antenatal care at St. Mary's Hospital Lacor. In Uganda, the clinical guideline advocates for the first antenatal care to be sought by a pregnant mother up to 20 weeks of gestation [19]. While all expectant mothers attending antenatal care at St. Mary's hospital Lacor were eligible, we included gestational ages of 16 to 24 weeks and those who gave written informed consent to participate in the study. Those whose pregnancies were less than 16 weeks were given a return date for the recruitment, while those above 24 weeks or had molar pregnancies, intrauterine fetal death and anencephaly were excluded.

We used consecutive sampling. We informed the mothers about the study during their morning health education meeting given to all mothers on arrival for prenatal care at the hospital. All the women who satisfied the inclusion criteria were approached and requested to provide informed consent. A research assistant administered questionnaire to capture their history and performed a physical examination. Some mothers were asked to give blood samples for full haemogram and liver and renal function tests. A few mothers (after the 1000^th^ mother) did not undergo laboratory tests for logistical reasons. An obstetrician performed the uterine artery Doppler sonography.

**Fig. 1:**
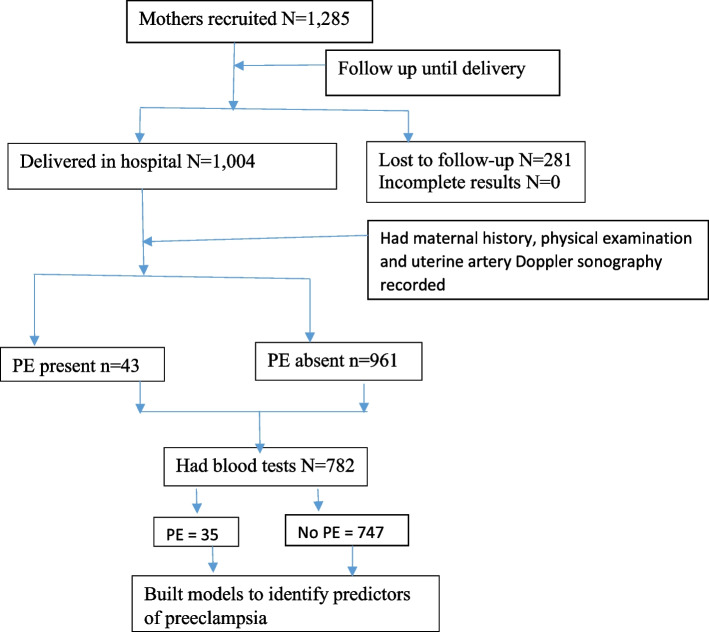
Flow chart of participants throughout the study

We recruited 1,285 pregnant mothers at 16–24 weeks from April 2019 to March 2020. All the mothers were of African ancestry at the end of the recruitment period. We followed up with the participants until September 2020. One thousand four (1,004) complete delivery records were obtained at the end of the study period. Seven hundred eighty-two (782) participants had laboratory blood tests (full haemogram, liver and renal function tests) done in addition to blood pressure readings, body mass index calculation and maternal history. Details are in Fig. 1.

The outcome was a combination of a blood pressure ≥ 140/90 mmHg and urine protein ≥  + 1 (pre-eclampsia) by delivery time. The data was pre-processed using Stata® version 15 and built models using RStudio version 4.1.3. Model 1 was built from second-trimester maternal history and physical examination findings, model 2 from the ultrasound and uterine artery Doppler indices, model 3 from a combination of maternal history, physical examination and ultrasound findings, model 4 from maternal laboratory tests, model 5 from a combination of laboratory tests with maternal history, and model 6 from the combination of all models.

We included all variables, did a univariate analysis and got unadjusted *p*-values for every variable collected. Afterwards, we had all variables with *p*-values ≤ 0.20 or known risk factors for pre-eclampsia in a logistic regression model and removed the non-statistically significant predictors step-wise. Finally, we retained the independent risk factors for pre-eclampsia and used them to build the models of choice, one with the least number of predictors with a higher AUC.

Because the incidence of pre-eclampsia was low (4.3%) [20], we generated synthetic balanced data using the ROSE package [21, 22] in RStudio by over-sampling pre-eclampsia and under-sampling non-preeclampsia. We got 383 (48.8%) for those diagnosed with pre-eclampsia and 399 (51.2%) for non-pre-eclampsia. Then, we evaluated the actual model performance against the ROSE-derived synthetic dataset using K-fold cross-validation in RStudio to obtain the AUC's accuracy, sensitivity, specificity and McFadden's pseudo R^2^. The variables were said to be independent risk factors for pre-eclampsia if their *p*-value < 0.05 in the model. The models, too, had a good fit if McFadden's value was between 0.2 – 0.4.

## Results

One thousand four (1,004) participants were used in a regression model to obtain the unadjusted relationship between the maternal history, clinical characteristics and pre-eclampsia. Seven hundred eighty-two (782) participants had additional laboratory blood tests (full haemogram, liver and renal function tests) and complete delivery records. So they were used to build the prediction models.

### Unadjusted relationship between the characteristics of pre-eclampsia

All variables with unadjusted *p*-values ≤ 0.20 or known risk factors are shown in Tables 1 and 2. All the variables with unadjusted *p*-values < 0.05 in the logistic regression model were independent risk factors for pre-eclampsia. In Table 1, the independent risk factors for pre-eclampsia were maternal history of pre-eclampsia, systolic hypertension, diastolic hypertension, prenatal hypertension, presence of an end-diastolic notch, pulsatility index > 1.34, and resistive index > 0.69.

**Table 1:** Unadjusted relative risk of maternal history and clinical characteristics with pre-eclampsia

Variable (*N* = 1,004)	IRR (95% CI)	*p*-value
Maternal age ≥ 35 (years)	1.53 (0.66—3.54)	0.3170
Para 1—2	1.51 (0.61—3.75)	0.3780
Nulliparity	2.30 (0.93—5.68)	**0.070**
Maternal history of pre-eclampsia	13.75 (7.44—25.42)	** < 0.001**
Age at menarche 13—16 years (years)	0.61 (0.33—1.16)	**0.1310**
Age at menarche > 16 years	0.47 (0.19—1.18)	**0.1070**
Body mass index 21.92—26.56 kg/m^2^	0.64 (0.27—1.49)	0.2990
Body mass index ≥ 26.56 kg/m^2^ (4th quadrant)	2.11 (0.99—4.48)	**0.054**
Systolic blood pressure Bp ≥ 140 mmHg	10.65 (4.29—26.41)	** < 0.001**
Diastolic blood pressure Bp ≥ 90 mmHg	5.36 (2.31—12.41)	** < 0.001**
hypertension at recruitment Bp ≥ 140/90 mmHg	12.48 (5.30—29.37)	** < 0.001**
Multiple pregnancies	4.95 (2.12—11.55)	** < 0.001**
Unilateral End diastolic notch	2.49 (1.13—5.49)	**0.0230**
Bilateral End diastolic notch	6.44 (3.38—12.25)	** < 0.001**
Average pulsatility index ≥ 1.34 (95th percentile)	6.43 (3.49—11.84)	** < 0.001**
Average Resistive index ≥ 0.69 (4th quadrant)	7.88 (4.33—14.35)	** < 0.001**

**Table 2:** Unadjusted relative risk of maternal laboratory tests with pre-eclampsia

Variable (*N* = 782)	IRR (95% CI)	*p*-value
Serum GGT (Gamma Glutamyl Transferase)0—30 iu	2.68 (0.83—8.64)	**0.0990**
Serum ALP (Alkaline phosphatase) < 98 iu	4.33 (1.81—10.35)	**0.0010**
Serum Albumin 3.5—4.1 g/dL	1.58 (0.59—4.22)	0.3610
Serum Albumin < 3.5 g/dL	2.82 (1.03—7.76)	**0.0450**
Serum Urea 11.0—44.0 mg/dL	3.64 (0.50—23.39)	0.2010
Serum Urea < 11.0 mg/dL	6.23 (0.72—54.29)	**0.0980**
Serum sodium 135.1—139.4 mmol/L (2nd—3rd quadrant)	0.97 (0.47—2.02)	0.9400
Serum sodium > 139.4 mmol/L (4th quadrant)	0.41 (0.13—1.30)	**0.1310**
Serum phosphorus 0.9—1.4 mg/dL (2nd—3rd quadrant)	2.22 (0.85—5.77)	**0.1030**
Serum phosphorus > 1.4 mg/dL (4th quadrant)	1.67 (0.56—5.03)	0.3590
Serum creatinine 0.8—1.2 µmol/L (2nd—3rd quadrant)	0.94 (0.45—1.94)	0.8600
Serum creatinine > 1.2 µmol/L (4th quadrant)	0.37 (0.10—1.31)	**0.1230**
Lymphocyte Count (0.8—4.0)*1000 cells/µl	0.37 (0.15—0.92)	**0.0330**
Lymphocyte Count (> 4.0)*1000 cells/µl	2.27 (0.68—7.53)	0.1820
Total White blood cell count (4.0—11.0)*1000 cells/µl	0.71 (0.28—1.81)	0.4780
Total White blood cell count (> 11.0)*1000 cells/µl	4.65 (1.40—15.50)	**0.0120**
Haematocrit 30.0—39.9%	1.01 (0.48—2.12)	0.9870
Haematocrit ≥ 40%	2.23 (0.79—6.26)	**0.1290**
Mean corpuscular volume (MCV) 80.0—100.0 fl	0.57 (0.29—1.11)	**0.0990**
Mean corpuscular volume (MCV) ≥ 100.0 fl	1.10 (0.16—7.85)	0.9210

In Table 2, the independent risk factors for pre-eclampsia were serum alkaline phosphatase (ALP) < 98 IU, serum albumin < 3.5 g/dL and total white blood cell count > 11,000 cells/µl. In addition, the lymphocyte count of 800 – 4,000 cells/µl was protective against pre-eclampsia.

### Risk factors and Prediction models for pre-eclampsia

In model 1 (Table 3), the predictors of pre-eclampsia were maternal age, parity, personal history of pre-eclampsia, body mass index, diastolic pressure, and multiple pregnancies. In addition, personal history of pre-eclampsia (aOR = 53.01, 95% CI 12.8—163.7, *p* = 0.000), nulliparity (aOR = 6.13, 95% CI 1.68—26.05, *p* = 0.009), diastolic hypertension ≥ 90 mmHg (aOR = 5.66, 95% CI 1.47—18.26, *P* = 0.006), multiple pregnancies (aOR = 5.16, 95% CI 1.07—18.45, *P* = 0.020), maternal age > 34 years (aOR = 4.69, 95% CI 1.28—16.36, *p* = 0.020), and body mass index ≥ 26.56 kg/m2 (aOR = 3.70, 95% CI 1.31—12.54, *p* = 0.021) were independent risk factors for pre-eclampsia.

**Table 3:** Showing model 1 shows the maternal history and physical examination for the prediction of pre-eclampsia

Variable	Adjusted Odds Ratio (95% CI)	*p*-value
Maternal age ≥ 35 years	4.69 (1.28—16.36)	0.020
Para 1—2	2.36 (0.72—8.71)	0.175
Nulliparity	6.13 (1.68—26.05)	0.009
Personal history of pre-eclampsia	53.01 (12.8—163.7)	** < 0.001**
BMI of 21.92—26.56 kg/m^2^	1.01 (0.33—3.51)	0.993
BMI of ≥ 26.56 kg/m^2^	3.70 (1.31—12.54)	0.021
Diastolic blood pressure ≥ 90 mmHg	5.66 (1.47—18.26)	0.006
Multiple pregnancies	5.16 (1.07—18.45)	0.020
Intercept	0.00 (0.00—0.02)	** < 0.001**

In model 2 (Table 4), the predictors of pre-eclampsia were end-diastolic notch and average pulsatility index. Bilateral end-diastolic notch (aOR = 3.71, 95% CI 1.30—9.81, *p* = 0.010) and average pulsatility index of ≥ 1.34 (aOR = 3.41, 95% CI 1.22—9.48, *p* = 0.018) were independent risk factors for pre-eclampsia.

**Table 4:** Shows model 2 shows uterine artery Doppler indices for the prediction of pre-eclampsia

Variable	Adjusted Odds Ratio (95% CI)	*p*-value
Unilateral end-diastolic notch	2.39 (0.92—5.78)	0.060
Bilateral end-diastolic notch	3.71 (1.30—9.81)	0.010
Average pulsatility index ≥ 1.34	3.41 (1.22—9.48)	0.018
Intercept	0.03 (0.01—0.04)	** < 0.001**

In model 3 (Table 5), the predictors of pre-eclampsia were maternal age, parity, personal history of pre-eclampsia, body mass index, diastolic pressure, multiple pregnancies and end-diastolic notch. In addition, personal history of pre-eclampsia (aOR = 36.88, 95% CI 8.40—178.40, *p* = 0.000), multiple pregnancies (aOR = 6.22, 95% CI 1.29—18.27, *p* = 0.015), maternal age > 34 years (aOR = 4.93, 95% CI 1.29—18.27, *p* = 0.017), bilateral end-diastolic notch (aOR = 4.40, 95% CI 1.68—11.29, *p* = 0.002), nulliparity (aOR = 4.39, 95% CI 1.19—19.54, *p* = 0.036), diastolic hypertension ≥ 90 mmHg (aOR = 4.39, 95% CI 1.06—15.21, *p* = 0.027), and body mass index ≥ 26.56 kg/m2 (aOR = 3.42, 95% CI 1.17—11.97, *p* = 0.034) were independent risk factors for pre-eclampsia.

**Table 5:** Shows model 3 shows a combination of maternal history and uterine artery Doppler indices for the prediction of pre-eclampsia

Variable	Odds Ratio (95% CI)	*p*-value
Maternal age Over 34 years	4.93 (1.29—18.27)	0.017
Para 1—2	2.13 (0.63—8.17)	0.244
Nulliparity	4.39 (1.19—19.54)	0.036
Personal history of pre-eclampsia	36.88 (8.40—178.40)	** < 0.001**
BMI of 21.92—26.56 kg/m^2^	1.03 (0.33—3.71)	0.957
BMI of ≥ 26.56 kg/m^2^ (4th quadrant)	3.42 (1.17—11.97)	0.034
Diastolic blood pressure ≥ 90 mmHg	4.39 (1.06—15.21)	0.027
Multiple pregnancies	6.22 (1.29—18.27)	0.015
Unilateral end-diastolic notch	2.39 (0.85—6.30)	0.083
Bilateral end-diastolic notch	4.40 (1.68—11.29)	0.002
Intercept	0.00 (0.00—0.02)	** < 0.001**

In model 4 (Table 6), the predictors of pre-eclampsia were white blood cell count, lymphocyte count, serum alkaline phosphatase, serum albumin, and serum urea. White blood cell count of > 11000cells/dl (aOR = 7.38, 95% CI 1.11—46.17, *p* = 0.033) and serum ALP < 98 IU/L (aOR = 5.84, 95% CI 1.78—16.39, *p* = 0.001) were independent risk factors for pre-eclampsia.

**Table 6:** Model 4 shows maternal laboratory characteristics for the prediction of pre-eclampsia

Variable	Odds Ratio (95% CI)	*p*-value
White cell count of (4.0—11.0)*10^3^	1.18 (0.40—4.25)	0.780
White cell count of (> 11.0)*103	7.38 (1.11—46.17)	0.033
Serum ALP (alkaline phosphatase) < 98 iu/L	5.84 (1.78—16.39)	0.001
Serum albumin 3.5–4.1 mg/dl	2.01 (0.74—6.60)	0.247
Serum albumin < 3.5 mg/dl	2.84 (0.97—9.67)	0.080
Serum urea 11.0—44.0 iu/L	4.30 (0.83—80.02)	0.158
Serum urea < 11.0 iu/L	8.00 (1.02—169.30)	0.074
Lymphocyte count of (0.8—4.0)*10^3^	0.29 (0.10—1.06)	0.041
Lymphocytes count of > 4.0*10^3^	1.30 (0.19—7.91)	0.705
Intercept	0.01 (0.00—0.06)	** < 0.001**

In model 5 (Table 7), the predictors of pre-eclampsia were maternal age, parity, personal history of pre-eclampsia, body mass index, diastolic pressure, white blood cell count, and serum ALP. Personal history of pre-eclampsia (aOR = 48.09, 95% CI 11.11—227.25, *p* = 0.000), serum ALP < 98 IU/L (aOR = 7.77, 95% CI 2.04—25.38, *p* = 0.001), diastolic hypertension ≥ 90 mmHg (aOR = 7.24, 95% CI 1.85—24.32, *p* = 0.002), white blood cell count > 11,000 cells/µl (aOR = 6.40, 95% CI 1.13—33.82, *p* = 0.028), nulliparity (aOR = 6.32, 95% CI 1.69—28.11, *p* = 0.010), body mass index of > 26.56 kg/m^2^ (aOR = 4.41, 95% CI 1.49—15.45, *p* = 0.012) and maternal age > 34 years (aOR = 3.88, 95% CI 1.01—14.32, *p* = 0.043) were independent risk factors for pre-eclampsia.

**Table 7:** Model 5 shows the maternal history and laboratory tests for the prediction of pre-eclampsia

Variable	Adjusted Odds Ratio (95% CI)	*p*-value
Maternal age Over 34 years	3.88 (1.01—14.32)	0.043
Para 1—2	2.62 (0.77—10.15)	0.140
Nulliparity	6.32 (1.69—28.11)	0.010
Personal history of pre-eclampsia	48.09 (11.11—227.25)	** < 0.001**
BMI of 21.92—26.56 kg/m^2^	1.06 (0.34—3.74)	0.923
BMI of ≥ 26.56 kg/m^2^	4.41 (1.49—15.45)	0.012
Diastolic blood pressure ≥ 90 mmHg	7.24 (1.85—24.32)	0.002
White cell count of (4.0—11.0)*10^3^	0.52 (0.18—1.74)	0.241
White cell count of > 11.0*10^3^	6.40 (1.13—33.82)	0.028
Serum ALP < 98.0 iu/L	7.77 (2.04—25.38)	0.001
Intercept	0.01 (0.001—0.03)	** < 0.001**

In model 6 (Table 8), the predictors of pre-eclampsia were maternal age, parity, personal history of pre-eclampsia, body mass index, diastolic pressure, white blood cell count, lymphocyte count, serum ALP and end-diastolic notch of the uterine arteries. Personal history of pre-eclampsia (aOR = 32.75, 95% CI 6.59—182.05, *p* = 0.000), serum ALP < 98 IU/L (aOR = 7.14, 95% CI 1.76—24.45, *p* = 0.003), diastolic hypertension ≥ 90 mmHg (aOR = 4.90, 95% CI 1.15—18.01, *p* = 0.022), bilateral end diastolic notch (aOR = 4.54, 95% CI 1.65—12.20, *p* = 0.003) and body mass index of ≥ 26.56 kg/m2 (aOR = 3.86, 95% CI 1.25—14.15, *p* = 0.027) were independent risk factors for pre-eclampsia.

**Table 8:** Model 6 shows maternal history, uterine artery Doppler indices, and laboratory tests for the prediction of pre-eclampsia

Variable	Adjusted Odds Ratio (95% CI)	*p*-value
Maternal age Over 34 years	3.88 (0.94—15.44)	0.056
Para 1—2	2.56 (0.73—10.62)	0.144
Nulliparity	4.25 (1.08—20.18)	0.051
Maternal history of pre-eclampsia	32.75 (6.59—182.05)	** < 0.001**
BMI of 21.92—26.56 kg/m^2^	1.09 (0.34—3.98)	0.888
BMI of ≥ 26.56 kg/m^2^	3.86 (1.25—14.15)	0.027
Diastolic blood pressure ≥ 90 mmHg	4.90 (1.15—18.01)	0.022
Unilateral end-diastolic notch	2.36 (0.81—6.39)	0.100
Bilateral end-diastolic notch	4.54 (1.65—12.20)	0.003
White cell count of (4.0—11.0) *10^3^ cells /µl	0.85 (0.24—3.49)	0.807
White cell count of > 11.0 *10^3^	8.43 (0.92—70.62)	0.050
Serum ALP < 98 iu/L	7.14 (1.76—24.45)	0.003
Lymphocyte count of (0.8—4.0) *10^3^	0.29 (0.08—1.22)	0.074
Lymphocytes count of > 4.0*10^3^	0.84 (0.09—6.96)	0.876
Intercept	0.01 (0.00—0.06)	** < 0.001**

### Evaluation of the models' performance

We evaluated the models using K (10) -fold cross-validation to obtain the accuracy, specificity, sensitivity, AUC and McFadden's pseudo R^2^. Details are in Table 9.

**Table 9:** Shows model performance evaluation using K-fold cross-validation

Model	Accuracy (%)	Sensitivity (%)	Specificity (%)	AUC (%)	McFadden's
Model 1 (History and physical exam)	66.6	82.7	49.9	78.4	**0.21**
Model 2 (Uterine artery Doppler indices)	68.8	73.7	63.7	71.4	0.09
Model 3 (Combination of models 1 and 2)	76.0	78.2	73.6	80.4	**0.25**
Model 4 (Maternal blood tests)	67.1	76.9	56.9	75.6	0.11
Model 5 (combination of models 1 and 4)	72.7	84.0	61.1	82.2	**0.26**
Model 6 (Combination of models 3 and 4)	77.0	80.2	73.6	84.9	**0.30**

Models 1, 3, 5 and 6 had a good fit with McFadden's pseudo-R-square between 0.2 and 0.4. Therefore they are helpful for the screening of pre-eclampsia in prenatal clinics

## Discussion

In this research, maternal history predictors of pre-eclampsia were maternal age, parity, personal history of pre-eclampsia, body mass index, diastolic pressure, and multiple pregnancies. They predicted pre-eclampsia with 66.6% accuracy, 82.7% sensitivity, and 78.4% AUC with a McFadden's pseudo R^2^ of 0.21. It can identify four out of five participants destined to develop pre-eclampsia. The low specificity of the model of close to 50% reduces the model's accuracy to 66%. This model is of good fit and can be used independently in prenatal clinics to screen for pre-eclampsia. In Ghana [23], predictors of pre-eclampsia were diastolic blood pressure, family history of hypertension in parents, history of pre-eclampsia in a previous pregnancy, nulliparity and obesity, with an AUC of the original model being 70% and 68% in the validation cohort. Gallo et al. [12] screened by maternal characteristics and mean arterial pressure (MAP) at a false-positive rate of 10%; their detection rate of total pre-eclampsia was 49.3%. In a systematic review by Al-Rubaie et al. [11], their detection rate was 76%.

With uterine artery Doppler indices, we predicted pre-eclampsia with over 68% accuracy and 71.4% AUC. Unfortunately, the model had a McFadden's pseudo R^2^ of 0.09 and was not a good fit. Therefore, this model cannot be used independently in prenatal clinics to screen for pre-eclampsia. That was way below Trudinger et al. [9], who predicted up to 90% of pre-eclampsia in Australia using an end-diastolic notch. Using a combination of maternal history, physical examination and uterine artery Doppler indices, we got an AUC of 80.4% with 76.0% accuracy. That is comparable to Pedroso et al. [24], who found a combination of uterine artery Doppler indices and maternal history predicted 75% of PE.

With laboratory blood tests, we predicted pre-eclampsia with 67.1% accuracy and 75.6% AUC with McFadden's pseudo R^2^ of 0.11. However, this model cannot be used independently in prenatal clinics to screen for pre-eclampsia. Jhee et al. [13] used a combination of serum urea, aspartate aminotransferase (AST), ALT, creatinine, and haemoglobin levels to predict pre-eclampsia with AUC above 57%. Yucel et al. [15] predicted pre-eclampsia using mean platelet volume (MPV) and plateletcrit (PCT) with AUC of 64.1% and 71.2%, respectively.

When we combined laboratory blood tests with maternal history, the accuracy improved to 72.7% with an AUC of 82.2% with a McFadden's pseudo R^2^ of 0.26. The combination of maternal history and uterine artery Doppler indices improved the accuracy to 76% with 80.4% AUC with a McFadden's pseudo R^2^ of 0.25. Combining maternal history, blood tests, and uterine artery Doppler indices (model 6) slightly improved the prediction accuracy to 77.0% and 80.2% sensitivity with an AUC of 84.9% with a McFadden's pseudo R^2^ of 0.30. All the combined models were of good fit and could be used independently in prenatal clinics to screen for pre-eclampsia.

Delic et al. [14] added uric acid, urea thrombocytes, hematocrit, AST and leukocytes into the logistic regression model and correctly classified 83.8% of patients with pre-eclampsia. That had a better detection rate than 57% in the UK [25]. A low level of serum ALP may signify a reduced viable mass of placental tissue in pregnancy [26, 27], which means a decreased surface area for the transfer of nutrients from mother to baby. This reduced surface area of the functional placenta may increase the number of placental infarcts and, eventually, placental debris released into the maternal circulation. In addition, increased levels of placental tissue in maternal circulation lead to maternal systemic inflammation [28]. That may result in endothelial injury, vasoconstriction and hypertension [29].

Duckit et al. [6], in a systematic review, found controlled cohort studies showing the risk of pre-eclampsia increased in women with a previous history of pre-eclampsia, multiple (twin) pregnancy, nulliparity, family history, raised blood pressure (diastolic ≥ 80 mm Hg) at booking, increased body mass index before pregnancy at booking, or maternal age ≥ 40. Antwi et al. [16] reviewed prediction models for pre-eclampsia between 2000 and 2019 and found diverse prediction accuracy ranging from 45 – 95% in the different regions of the world. The other prediction rates could explain the differences in the populations studied or the test techniques and the ultrasound machines used. Our models seem within acceptable accuracy, although the whole study population was at high risk. These models will ease the identification of high-risk mothers and referral to specialists' healthcare providers. That may, in turn, contribute to reducing maternal mortality and morbidity in the community.

### Strength of the study

This is a baseline study in northern Uganda and could open ways for further research on pre-eclampsia in this community.

### Weakness of the study

There were many losses to follow-ups, which could have skewed the results differently. The data collection period (April 2019 to September 2020) coincided with part of the covid -19 lockdowns in Uganda. Many mothers could not come to the hospital and may have delivered from home or in smaller health units near their homes. Patients were not motivated by transport refunds or covering hospital bills. The government hospital (Gulu regional referral hospital) was only 6 km away, offering free prenatal and delivery services. That could have affected the return of those who could not afford the hospital bills. Future studies could find ways of motivating mothers to deliver in hospitals.

### What is already known about this topic

It is known that women of African descent are more at risk of pre-eclampsia than other communities [5–7]. It is also known that in prenatal clinics where pre-eclampsia is predicted, early diagnosis and appropriate treatment are made to save lives [30–32].

### What new knowledge the study adds

The new knowledge added through this study is that the incidence of pre-eclampsia in this black community is comparable to global estimates of pre-eclampsia [33]. The predictors of pre-eclampsia are also similar to other communities. We also predicted pre-eclampsia using a full haemogram, liver and renal function tests, uterine artery Doppler sonography, and maternal history. However, the Doppler indices percentiles differ when compared to the global north.

### Implication for practice

The prediction models can be adapted for use in prenatal clinics to screen mothers for the prediction of pre-eclampsia. In addition, data from such clinics can be used to validate the models.

## Conclusions

Predictors of pre-eclampsia in the low resource setting of northern Uganda are maternal age ≥ 35 years, nulliparity, maternal history of pre-eclampsia, body mass index, diastolic pressure, white blood cell count, lymphocyte count, serum alkaline phosphatase and end-diastolic notch of the uterine arteries. Prenatal clinics without any ultrasound scans or laboratory can adequately predict pre-eclampsia with up to 66.6% accuracy and 78.4% AUC. However, clinics with Doppler ultrasound and laboratory tests can use maternal history either with ultrasound or laboratory blood tests or both. That will improve the prediction AUC to over 80%.

### Recommendation

We built the models based on data from one health facility, and most of the respondents lived in one region of Uganda. Therefore, we recommend that the models be further validated with datasets from other areas of the country to scale up the use.

## Data Availability

Dr Silvia Awor or Makerere University Directorate of Research and Graduate training has the dataset.
